# Cardiac power parameters during hypovolemia, induced by the lower body negative pressure technique, in healthy volunteers

**DOI:** 10.1186/s12871-016-0195-0

**Published:** 2016-06-30

**Authors:** Audun Eskeland Rimehaug, Ingrid Elise Hoff, Lars Øivind Høiseth, Jonny Hisdal, Petter Aadahl, Idar Kirkeby-Garstad

**Affiliations:** 1 Department of Anesthesiology and Intensive care, St Olav Trondheim University Hospital, Trondheim, Norway; 2 Department of Circulation and Medical Imaging, NTNU, Norwegian University of Science and Technology, Trondheim, Norway; 3 Circulation research group Trondheim (CIRCUT), Trondheim, Norway; 4 Norwegian Air Ambulance Foundation, Drøbak, Norway; 5 Department of Anesthesiology, Oslo University Hospital, Oslo, Norway; 6 Faculty of Medicine, University of Oslo, Oslo, Norway; 7 Department of Vascular Medicine, Oslo University Hospital, Oslo, Norway

**Keywords:** Cardiac power, Hypovolemia, Critical care, Lower body negative pressure, Vascular impedance

## Abstract

**Background:**

Changes in cardiac power parameters incorporate changes in both aortic flow and blood pressure. We hypothesized that dynamic and non-dynamic cardiac power parameters would track hypovolemia better than equivalent flow- and pressure parameters, both during spontaneous breathing and non-invasive positive pressure ventilation (NPPV).

**Methods:**

Fourteen healthy volunteers underwent lower body negative pressure (LBNP) of 0, −20, −40, −60 and −80 mmHg to simulate hypovolemia, both during spontaneous breathing and during NPPV. We recorded aortic flow using suprasternal ultrasound Doppler and blood pressure using Finometer, and calculated dynamic and non-dynamic parameters of cardiac power, flow and blood pressure. These were assessed on their association with LBNP-levels.

**Results:**

Respiratory variation in peak aortic flow was the dynamic parameter most affected during spontaneous breathing increasing 103 % (*p* < 0.001) from baseline to LBNP −80 mmHg. Respiratory variation in pulse pressure was the most affected dynamic parameter during NPPV, increasing 119 % (*p* < 0.001) from baseline to LBNP −80 mmHg. The cardiac power integral was the most affected non-dynamic parameter falling 59 % (*p* < 0.001) from baseline to LBNP −80 mmHg during spontaneous breathing, and 68 % (*p* < 0.001) during NPPV.

**Conclusions:**

Dynamic cardiac power parameters were not better than dynamic flow- and pressure parameters at tracking hypovolemia, seemingly due to previously unknown variation in peripheral vascular resistance matching respiratory changes in hemodynamics. Of non-dynamic parameters, the power parameters track hypovolemia slightly better than equivalent flow parameters, and far better than equivalent pressure parameters.

## Background

Detecting hypovolemia and predicting fluid responsiveness remain difficult tasks in emergency medicine, intensive care and the operating theatre [1–5], particularly during spontaneous breathing [6, 7]. Both over- and underestimating the need for fluid resuscitation could have devastating effects. Cardiac power (PWR), measured in Watts, is calculated as the continuous product of aortic pressure and aortic flow. As a consequence reductions in both pressure and flow due to hypovolemia will be incorporated in cardiac power, which theoretically should make cardiac power parameters able to track hypovolemia better than the two factors separately. We are developing a minimally invasive system for beat-by-beat measurement of cardiac power [8], soon ready for clinical research regarding possible applications including detection of hypovolemia. In this study we have analyzed previously recorded data from healthy volunteers using a laboratory system [9] with lower body negative pressure (LBNP) to simulate hypovolemia [10], to consider the potential use of cardiac power parameters in hemodynamically unstable patients.

The cardiac power parameters we chose to study were maximal cardiac power (PWR_max_), the cardiac power integral (PWR-integral), and cardiac power output (CPO). PWR_max_ is the maximal value of cardiac power in each cardiac cycle. The PWR-integral represents the total energy measured in Joules transferred from the heart to the aorta per heartbeat, and is calculated as the area under the cardiac power curve per cardiac cycle. CPO represents mean cardiac power in Watts, excluding the oscillatory (also known as pulsatile) power consumed by the pulsatile movement of blood [11]. It is is calculated as CPO = MAP * CO/451, where MAP is mean arterial pressure in mmHg, and CO is cardiac output in l/min.

We wanted to compare cardiac power parameters to equivalent pressure- and flow parameters. We have made a distinction between dynamic and non-dynamic parameters. By dynamic parameters we refer to changes in hemodynamic measures in response to a defined perturbation [12, 13]. In this study it denotes respiratory variation in hemodynamic variables. By non-dynamic parameters we refer to selected standard hemodynamic measures such as cardiac output (CO), stroke volume (SV), peak aortic flow, and mean arterial pressure (MAP).

As mentioned, since cardiac power is a function of both blood pressure and blood flow, one could expect cardiac power parameters to be more affected by hypovolemia than each of the two factors separately. However, changes in the shape and phase of the pressure and flow curve as a result of hypovolemia could affect the impact on power parameters, necessitating this study. We chose to investigate the power parameters both during spontaneous breathing and non-invasive positive pressure ventilation (NPPV). During spontaneous breathing respiratory variation in blood pressure and aortic flow have insufficient sensitivity for detecting hypovolemia, but power parameters may be more affected as a result of incorporating respiratory variation in both flow and pressure. During non-invasive positive pressure ventilation (NPPV) respiratory variation in pulse pressure has previously shown significant results [9], respiratory variation in power parameters may however track hypovolemia even better.

Our first hypothesis regarding dynamic parameters was that respiratory variations in cardiac power parameters would incorporate respiratory variations in pressure and flow, and therefore track hypovolemia better than respiratory variations in stroke volume (Δ SV) and pulse pressure (Δ PP) [14–17]. Our second hypothesis was that non-dynamic parameters of cardiac power would track hypovolemia better than existing non-dynamic parameters, in particular SV, which has shown the best ability to track hypovolemia in previous studies [9]. Both hypotheses were tested during spontaneous breathing and during non-invasive positive pressure ventilation (NPPV).

## Methods

Approval to reanalyze existing data was given by the Norwegian Regional Ethics Committee South East (REK sør-øst 2015/432). Approval of the original experiment was given in 2009 (REK sør-øst 2009/2180). The paragraphs “Subject inclusion”, “Experimental protocol” and “Data acquisition” below describe the original experiment, while the remaining paragraphs in the methods section describe the analysis specifically for this study.

### Subject inclusion

Written informed consent to participate and allow publication of results was obtained from 14 healthy volunteers (7 male, 7 female, aged 28 ± 7 years, height 177 ± 10 cm, and weight 71 ± 13 kg (mean ± SD)), from which 13 completed sufficient of the protocol to be included in analysis. The subjects were instructed to refrain from alcohol or caffeinated drinks 24 h prior to participation. Pregnant women and subjects using cardiovascular medication were not included. The number of subjects was chosen based on similar LBNP studies investigating other hemodynamic parameters.

### Experimental protocol

Subjects were in the supine position during the experiments, which were performed in room temperature. LBNP was applied by a custom made LBNP chamber previously described [18], and induced by stepwise suction of air out of the chamber. After baseline measurements, subjects underwent consecutive LBNP-pressures of −20, −40, −60, and −80 mmHg. Each level was kept for 4.5 min. After a minimum of 15 min rest, the procedure was repeated with NPPV. NPPV was applied via a face mask connected to a Dräger Evita 4 ventilator (Dräger Medizintechnik GmbH, Lübeck, Germany) in volume control mode with, tidal volume 10 mL/kg ideal weight, positive end-expiratory pressure (PEEP) = 0 cm H_2_O, fraction of inspired oxygen 0.21, and respiratory frequency of 10–12/min. Volume control mode with intermittent positive pressure ventilation (IPPV) was chosen because it minimized spontaneous breathing activity and improved compliance with the ventilator. See also “Limitations of the study”. Spontaneous breathing and mask leakage were further minimized by thorough mask adjustment and by ensuring compliance with the ventilation mode before data recordings. The protocol was discontinued if one of the following events occurred: systolic blood pressure (SBP) <70 mmHg, a sudden decrease in SBP ≥15 mmHg, a decrease in heart rate (HR) ≥15 beats/min, dizziness, sweating, or nausea.

### Data acquisition

**Fig. 1:**
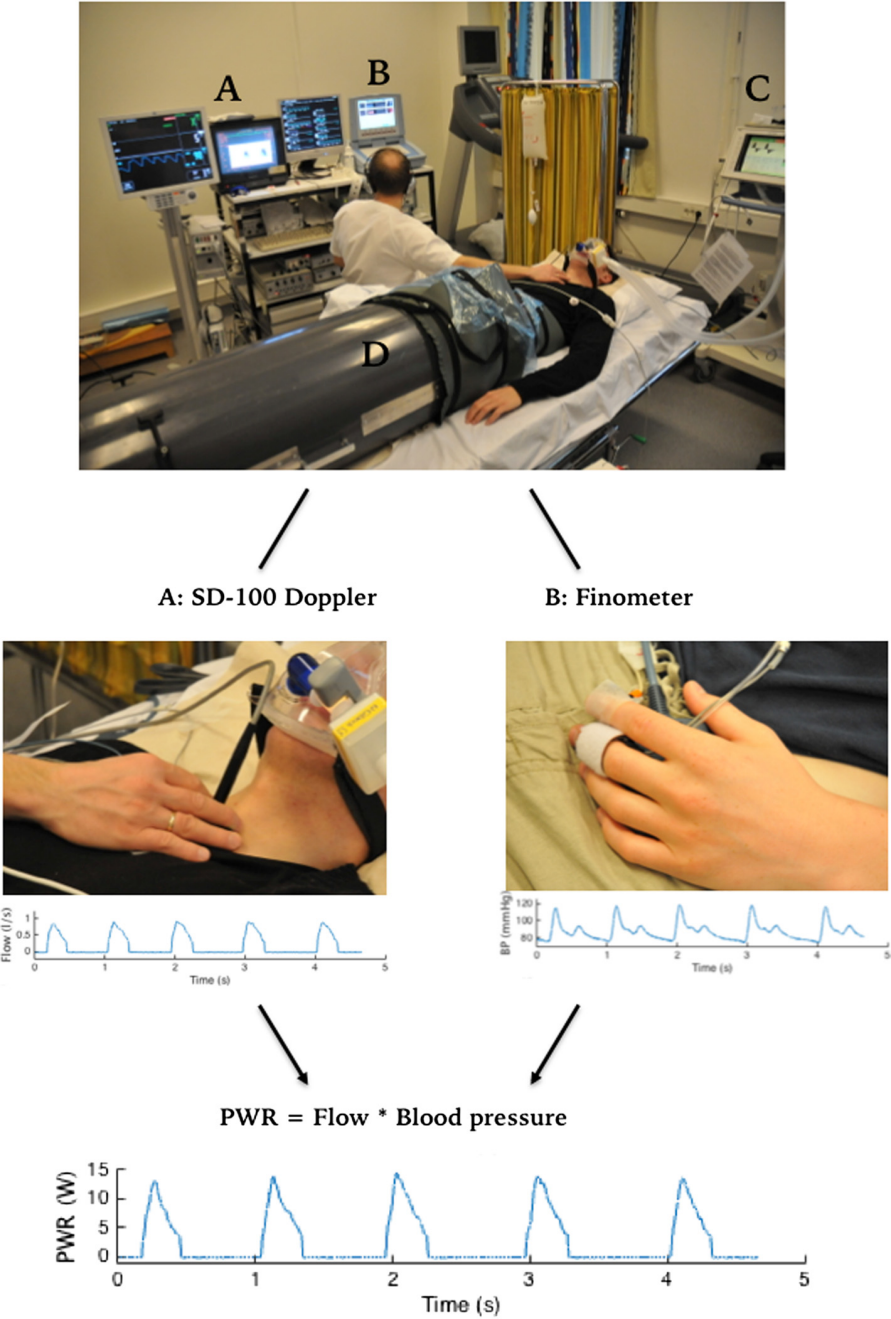
Illustration of setup and cardiac power calculation. Top image depicts the setup with the GE Vingmed SD-100 Doppler monitor (A), the Finometer blood pressure monitor (B), the Dräger Evita 4 ventilator (C) and the in-house LBNP chamber (D). The traces from the suprasternal Doppler flow and the Finometer blood pressure measurement were recorded by in-house software, and used as basis for calculation of a cardiac power trace (PWR) in Matlab

Data were recorded over the total duration of each LBNP-level. Data from all completed LBNP-levels are included in the analysis. At each LBNP-level calculations were made from data sampled over 10 consecutive respiratory cycles without arrhythmia. Respiratory movements were recorded with a custom-made air flowmeter. Continuous arterial pressure was obtained noninvasively at heart level from the left third finger (Finometer, FMS Finometer Medical Systems BV, Amsterdam, The Netherlands). Aortic flow was obtained continuously with suprasternal Doppler (SD-100, GE Vingmed Ultrasound, Horten, Norway) by an experienced operator. An angle of 20 ^o^ and a diameter of the aortic valve of 20 mm were assumed in the calculation of SV from the velocity-time integrals. The assumption of 20 mm should be appropriate in healthy volunteers [19], and assuming a fairly constant diameter in each individual [20] relative changes in aortic flow and therefore cardiac power due to LBNP will be uncovered regardless of the error this diameter estimate introduces. Heart rate (HR) was obtained from a standard 3-lead electrocardiogram (ECG). All signals were sampled at 300 Hz. The setup and calculation of cardiac power is illustrated in Fig. 1.

### Data analysis

All data analysis was performed using Matlab (R2013a, MathWorks, Natick, MA, USA). The cardiac power trace was calculated by multiplying aortic flow trace from the suprasternal Doppler with continuous arterial pressure trace from the left third finger, using a transfer function which corrected the time delay of the peripheral pressure compared with aortic flow by synchronizing the systolic upstroke of the two signals. This shifted the pressure curve approximately 5 milliseconds.

Ten consecutive respiratory cycles and the corresponding hemodynamic data were identified and stored for each subject in each LBNP condition, for spontaneous breathing and NPPV independently. The aortic flow and peripheral pressure curves were extracted for these intervals of ten respiratory cycles, and a cardiac power curve was calculated as the product of the flow and pressure curve. PWR_max_ was calculated as the maximal value of cardiac power in each cardiac cycle. The PWR-integral was calculated as the time integral of the power curve in each cardiac cycle, graphically represented as the area under the cardiac power curve in one cardiac cycle. Cardiac Power Output (CPO) was calculated as SV x HR x MAP/451 [21].

All the non-dynamic parameters were calculated per cardiac cycle, and then averaged over one respiratory cycle to create one data point. The dynamic parameters were calculated as the difference between the maximal and minimal value of each hemodynamic parameter within one respiratory cycle, divided by their mean value, thus representing the respiratory variations [22]. Consequently, each respiratory cycle gave one data point both for the dynamic and the non-dynamic parameters.

### Statistical analysis

Statistical calculations were performed in SPSS ver 20 (SPSS Inc, Chicago, IL, USA). Mean and standard deviation of each hemodynamic parameter was calculated for each level of LBNP, for NPPV and spontaneous breathing separately. To determine if the change in the hemodynamic parameter at each level of LBNP was significant compared to the baseline value, the data were analyzed in a linear mixed model with LBNP as a fixed factor and subject as a random factor. This was done for NPPV and spontaneous breathing separately. The linear mixed model was used to maximize statistical power by utilizing all data despite missing observations following the premature termination of the LBNP-protocol in some subjects and to account for the dependency of observations within subjects. Where the change in the hemodynamic parameter was significant at a level of *p* < 0.05, the change in percentage from the baseline value was calculated.

### Impedance analysis

A finding of a phase difference between respiratory variation in pressure and flow described in “Discussion” prompted an impedance analysis, which was not part of the original study. Arterial impedance is a frequency dependent analysis of the opposition to blood flow, which provides a more complex and complete assessment of the relation between blood flow and pressure than when using peripheral resistance alone [23]. Particularly interesting for our study, impedance analysis can determine components of vascular resistance arising from peripheral and proximal vasculature separately.

**Fig. 2:**
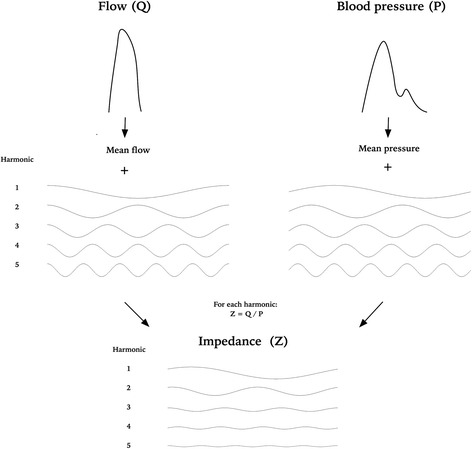
Illustration of impedance analysis by Fourier transformation. Pressure and flow from the aorta are converted into a series of sine waves using Fourier transformation. For each harmonic the corresponding waves of pressure and flow are related to calculate an amplitude and phase angle of each harmonic of the impedance. Only the first five harmonics are included in the figure for illustative purposes

The concept of impedance analysis is well described in specific textbooks on hemodynamics [11, 24]. Our approach used the fast Fourier transform function (fft) in Matlab to deconstruct the pressure and flow signal of each cardiac cycle into fundamental sine waves, as illustrated in Fig. 2. These sine waves were used to calculate the sine waves of the arterial impedance during each cardiac cycle, described by their amplitudes |Zn| and phase angles ϕ_n_. Lower harmonics, that is lower values of n, correspond to lower frequencies and therefore impedance arising from distal vasculature, while the higher harmonics arise from proximal vasculature [11, 24, 25]. We then graphically evaluated how these impedance amplitudes varied through the respiratory cycle.

## Results

### Dynamic parameters

**Fig. 3:**
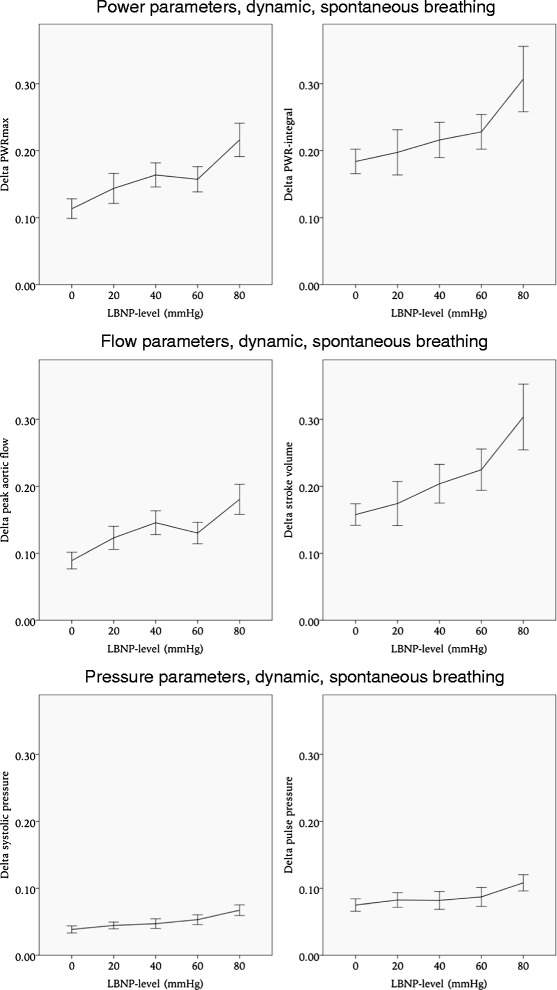
Dynamic parameters during spontaneous breathing. Error bars represent 95 % confidence interval. LBNP: lower body negative pressure

During spontaneous breathing the dynamic parameters that were most affected by LBNP were respiratory variations in maximal aortic flow (Δ peak aortic flow) and PWR_max_ (Δ PWR_max_). As presented in Table 1 and Fig. 3, Δ peak aortic flow increased by 103 % (*p* < 0.001), and Δ PWR_max_ increased by 91 % (*p* < 0.001) from baseline to LBNP 80 mmHg. The increase in Δ peak aortic flow and Δ PWR_max_ was significant at all levels of LBNP to a *p*-value < 0.05. Δ PWR-integral, Δ SV, Δ SBP and Δ PP had smaller changes associated with LBNP, details are presented in Table 1 and Fig. 3.

**Table 1:** Dynamic parameters. Changes in respiratory variation in power, flow and pressure parameters, both during spontaneous breathing and NPPV

Power parameters, dynamic								
	Delta PWRmax (*10-2)				Delta PWR-integral (*10-2)			
	Spontaneous breathing		NPPV		Spontaneous breathing		NPPV	
	Mean (sd)	Change	Mean (sd)	Change	Mean (sd)	Change	Mean (sd)	Change
Baseline	11.3 (7.0)		16.4 (7.6)		18.3 (8.9)		20.8	
20 mmHg	*14.3 (11.6)	+27 %	16.2 (6.9)		19.7 (17.6)		20.3	
40 mmHg	**16.3 (9.4)	+44 %	16.4 (7.7)		21.6 (13.7)		*26 (15.4)	+25 %
60 mmHg	*15.7 (8.9)	+39 %	**20.6 (9.0)	+26 %	22.8 (12.3)		*25.9 (14)	+25 %
80 mmHg	**21.6 (11.8)	+91 %	**27.1 (9.7)	+65 %	**30.7 (23.2)	+68 %	**39.9 (22.2)	+92 %
Flow parameters, dynamic								
	Delta peak aortic flow (*10-2)				Delta stroke volume (*10-2)			
	Spontaneous breathing		NPPV		Spontaneous breathing		NPPV	
	Mean (sd)	Change	Mean (sd)	Change	Mean (sd)	Change	Mean (sd)	Change
Baseline	8.9 (6.0)		14 (7.2)		15.8 (7.8)		20.1 (11.7)	
20 mmHg	**12.3 (9.0)	+38 %	14.5 (7.3)		17.4 (17.1)		19.2 (11.4)	
40 mmHg	**14.6 (9.2)	+64 %	14.6 (7.3)		20.4 (14.9)		*25.3 (16.6)	+26 %
60 mmHg	**13 (7.6)	+46 %	15.4 (8.3)		*22.5 (14.6)	+42 %	*25.9 (14.0)	+29 %
80 mmHg	**18.1 (10.7)	+103 %	**20.3 (7.9)	+45 %	**30.4 (23.3)	+92 %	**40.6 (23.7)	+102 %
Pressure parameters, dynamic								
	Delta systolic pressure (*10-2)				Delta pulse pressure (*10-2)			
	Spontaneous breathing		NPPV		Spontaneous breathing		NPPV	
	Mean (sd)	Change	Mean (sd)	Change	Mean (sd)	Change	Mean (sd)	Change
Baseline	3.8 (2.6)		4.3 (2.6)		7.5 (4.5)		6.8 (6.0)	
20 mmHg	4.5 (2.6)		4.7 (2.5)		8.2 (5.6)		8 (4.9)	
40 mmHg	4.7 (3.8)		*5.5 (2.6)	+28 %	8.2 (6.9)		*8.9 (5.0)	+31 %
60 mmHg	*5.3 (3.5)	+39 %	*7.3 (3.8)	+70 %	8.7 (6.7)		**13.1 (8.0)	+93 %
80 mmHg	**6.7 (3.8)	+76 %	**10 (4.3)	+133 %	**10.8 (5.8)	+44 %	**14.9 (6.5)	+119 %

*Significant to a level of *p* < 0.05

**Significant to a level of *p* < 0.001

**Fig. 4:**
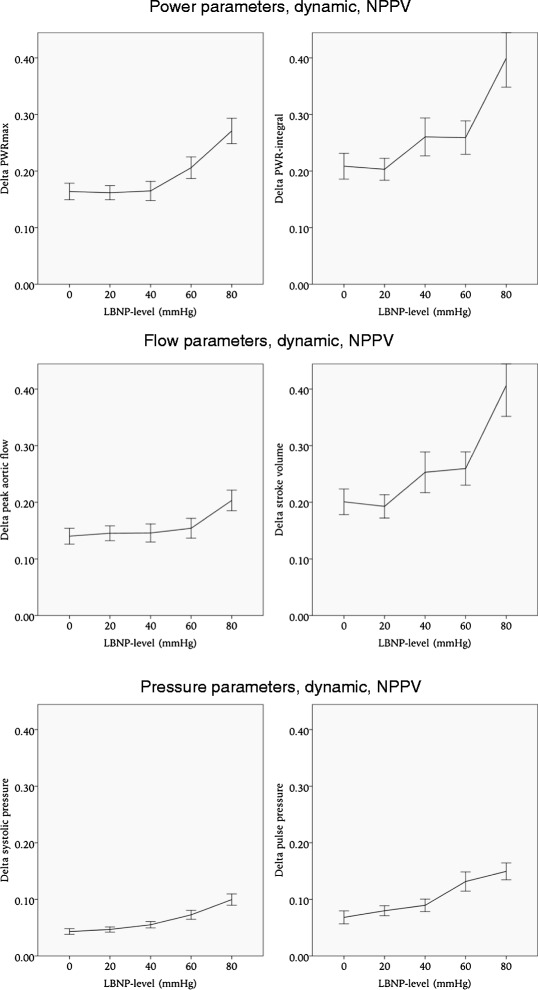
Dynamic parameters during non-invasive positive pressure ventilation (NPPV). Error bars represent 95 % confidence intervals. LBNP: lower body negative pressure

During NPPV the dynamic parameter that was most affected by LBNP was Δ PP, increasing by 119 % at LBNP 80 mmHg compared to baseline. The increase in Δ PP was significant at LBNP 40 mmHg with a *p*-value < 0.05, and at LBNP 60 mmHg and 80 mmHg with a *p*-value < 0.001. Δ PWR_max_, Δ PWR-integral, Δ peak aortic flow, Δ SV and Δ SBP were less affected by LBNP, details are presented in Table 1 and Fig. 4.

### Non-dynamic parameters

**Fig. 5:**
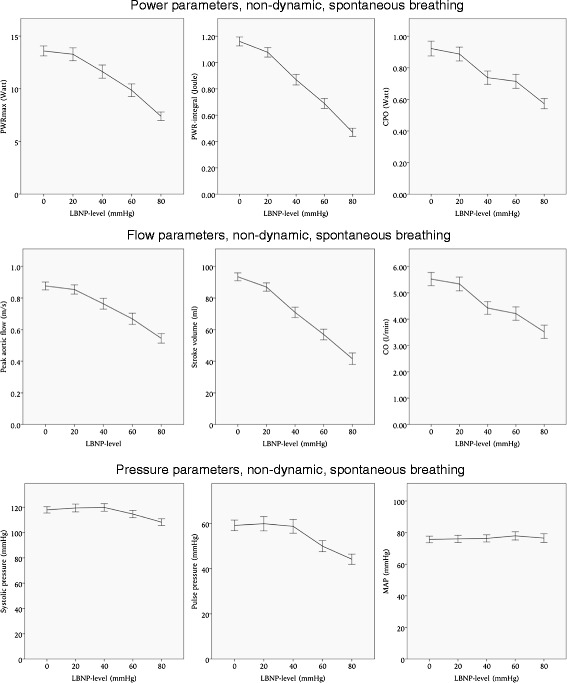
Non-dynamic hemodynamic parameters during spontaneous breathing. Error bars represent 95 % confidence intervals. LBNP: lower body negative pressure

During spontaneous breathing the PWR-integral was the non-dynamic parameter most affected by LBNP, with a 59 % reduction from baseline to LBNP 80 mmHg, while SV had a 55 % reduction. The reductions in the PWR-integral and SV compared to baseline were significant to *p* < 0.001 at all levels of LBNP. Reductions in PWR_max_, CPO, peak aortic flow, CO, SBP, PP and MAP were smaller and less significant, details are presented in Table 2 and Fig. 5.

**Table 2:** Non-dynamic parameters. Reduction in non-dynamic power, flow and pressure, both during spontaneous breathing and NPPV

Power parameters, non-dynamic												
	PWRmax (Watt)				PWR-integral (Joule)				CPO (Watt)			
	Spontaneous breathing		NPPV		Spontaneous breathing		NPPV		Spontaneous breathing		NPPV	
	Mean (sd)	Change	Mean (sd)	Change	Mean (sd)	Change	Mean (sd)	Change	Mean (sd)	Change	Mean (sd)	Change
Baseline	13.6 (2.8)		13.3 (2.4)		1.03 (0.14)		1.01 (0.16)		0.92 (0.23)		0.95 (0.21)	
20 mmHg	13.3 (3.2)		**11.8 (3.1)	−11 %	**0.96 (0.17)	−6.9 %	**0.84 (0.19)	−17 %	0.89 (0.23)		**0.81 (0.20)	−15 %
40 mmHg	**11.6 (3.2)	−15 %	**10.2 (3.5)	−23 %	**0.77 (0.19)	−25 %	**0.66 (0.20)	−34 %	**0.73 (0.22)	−21 %	**0.73 (0.25)	−23 %
60 mmHg	**9.9 (2.8)	−27 %	**8.8 (3.3)	−34 %	**0.69 (0.19)	−41 %	**0.51 (0.19)	−50 %	**0.71 (0.21)	−23 %	**0.64 (0.27)	−33 %
80 mmHg	**7.4 (1.9)	−46 %	**6.7 (3.0)	−50 %	**0.42 (0.13)	−59 %	**0.32 (0.11)	−68 %	**0.57 (0.15)	−38 %	**0.48 (0.18)	−49 %
Flow parameters, non-dynamic												
	Aorta maxflow (m/s)				Stroke volume (ml)				CO (L/min)			
	Spontaneous breathing		NPPV		Spontaneous breathing		NPPV		Spontaneous breathing		NPPV	
	Mean (sd)	Change	Mean (sd)	Change	Mean (sd)	Change	Mean (sd)	Change	Mean (sd)	Change	Mean (sd)	Change
Baseline	0.88 (0.12)		0.85 (0.15)		83.0 (10.6)		77.9 (13.4)		4.91 (1.08)		**4.85 (1.33)	
20 mmHg	0.85 (0.15)		*0.78 (0.16)	−8.2 %	*77.3 (12.1)	−6.9 %	**67.6 (16.4)	−13 %	4.74 (1.2)		**4.26 (0..99)	−12 %
40 mmHg	**0.76 (0.18)	−14 %	**0.66 (0.21)	−22 %	**63.0 (15.3)	−24 %	**52.3 (17.6)	−33 %	**3.93 (1.11)	−20 %	**3.64 (1.28)	−25 %
60 mmHg	**0.67 (0.17)	−24 %	**0.62 (0.18)	−27 %	**50.7 (14.1)	−39 %	**44.2 (13.7)	−43 %	**3.74 (1.08)	−24 %	**3.48 (1.33)	−28 %
80 mmHg	**0.55 (0.14)	−38 %	**0.53 (0.14)	−38 %	**37.0 (15.5)	−55 %	**30.4 (11.9)	−61 %	**3.13 (1.06)	−36 %	**2.89 (1.01)	−40 %
Pressure parameters, non-dynamic												
	Systolic pressure (mmHg)				Pulse pressure (mmHg)				MAP (mmHg)			
	Spontaneous breathing		NPPV		Spontaneous breathing		NPPV		Spontaneous breathing		NPPV	
	Mean (sd)	Change	Mean (sd)	Change	Mean (sd)	Change	Mean (sd)	Change	Mean (sd)	Change	Mean (sd)	Change
Baseline	118 (12.2)		123 (17.3)		59.2 (11.4)		61.5 (10.8)		75.7 (10.1)		80.2 (16.1)	
20 mmHg	119 (16.2)		121 (22.2)		59.9 (16.5)		59.7 (15.4)		76.0 (11.7)		78.0 (18.2)	
40 mmHg	120 (15.6)		124 (21.6)		58.8 (15.8)		*56.1 (18.3)	−9 %	76.3 (11.7)		82.1 (18.7)	
60 mmHg	115 (13.4)		*114 (27.0)	−7.3 %	**50 (11.7)	−16 %	**50.2 (19.3)	−18 %	77.9 (12.3)		76.8 (21.4)	
80 mmHg	**108 (−12.6)	−8.4 %	**98 (27.7)	−20 %	**44.2 (10.7)	−25 %	**40.3 (18.1)	−34 %	76.5 (13.0)		**69.7 (22.8)	−13.1 %

*Significant to a level of *p* < 0.05

**Significant to a level of *p* < 0.001

**Fig. 6:**
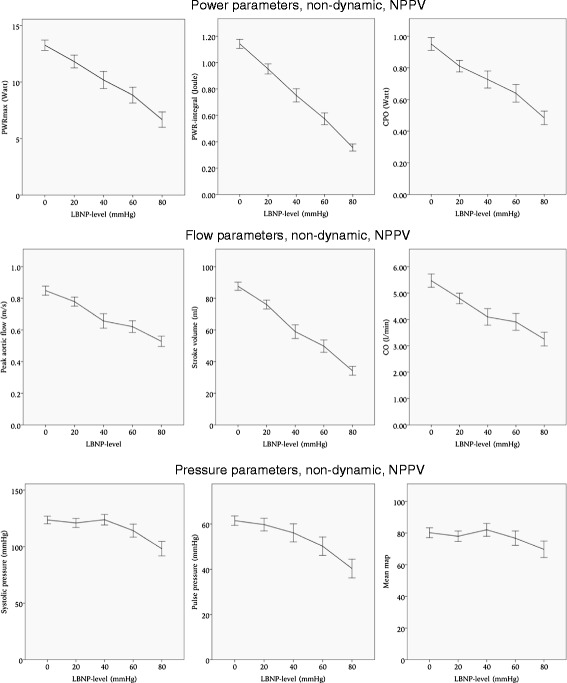
Non-dynamic hemodynamic parameters during non-invasive positive pressure ventilation (NPPV). Error bars represent 95 % confidence intervals. LBNP: lower body negative pressure

During NPPV the non-dynamic parameters most affected by LBNP were the PWR-integral and SV, as in spontaneous breathing. The PWR-integral was reduced by 68 % (*p* < 0.001) from baseline to LBNP 80 mmHg, while SV was reduced by 61 % (*p* < 0.001). Details are presented in Table 2 and Fig. 6.

## Discussion

The main finding in this study was that dynamic cardiac power parameters somewhat surprisingly were less able to track hypovolemia than equivalent dynamic flow- and pressure parameters both during spontaneous ventilation and during NPPV_._ The PWR-integral was the best non-dynamic parameter both during spontaneous breathing and during NPPV. However, SV tracked hypovolemia almost as well as the PWR-integral.

### Dynamic parameters

**Fig. 7:**
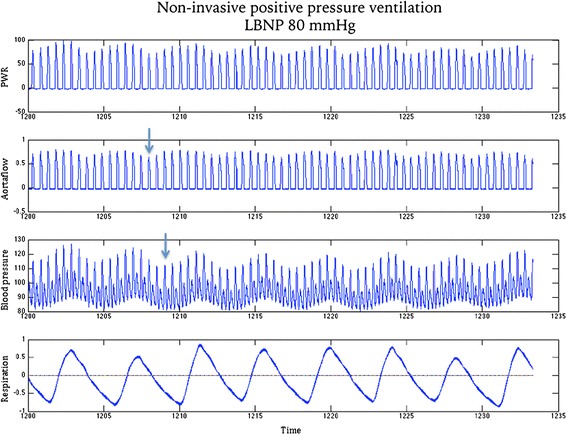
The PWR trace, which is the product of the synchronized aortic flow trace and the blood pressure trace. The arrows indicate that respiratory variation in aortic flow is 2–3 heart cycles ahead of respiratory variation in blood pressure

The first hypothesis, that respiratory variations in PWR parameters would be stronger than respiratory variations in flow- and pressure parameters, is not in agreement with our observations. The reason seems to be that respiratory variations in flow and pressure are out of phase, as illustrated in Fig. 7. Respiratory variation in flow seems to be 2–3 cardiac cycles ahead of pressure variation, which would lead to these variations partially cancelling each other out when pressure and flow are combined to calculate the PWR trace. This observation is consistent across individuals and LBNP-levels, and likely explains why Δ PWR_max_ and Δ PWR-integral did not follow LBNP-levels better.

The finding that respiratory variation in flow lags respiratory variation in pressure by 2–3 cardiac cycles indicates cyclic changes in impedance, the vascular opposition to blood flow. We retrospectively performed an impedance analysis by Fourier transformation [23, 24, 26] using Matlab, to see which part or parts of the vascular system was responsible for these cyclic changes. The systemic vascular resistance (SVR), also described as Z0, and the first impedance harmonic amplitude |Z1| [23] showed signs of cyclic changes following respiration in several of the research subjects, indicating cyclic changes in the peripheral vascular resistance matching respiratory variation in hemodynamic parameters. The characteristic impedance amplitude Zc and f0, the frequency where the impedance phase first crosses 0 [23], did not follow the respiratory cycle, indicating that impedance of the proximal vasculature and reflections did not play a part in the cyclic changes of the total impedance. These findings were however not convincing in all subjects, typically the subjects who cooperated best with the ventilator showed these signs most clearly. In the literature we have found indications of cyclic variation in peripheral resistance with frequencies up to 0.1 Hz [27]. However, cyclic variation in hemodynamic parameters matching respiration will have significantly higher frequencies. We suggest further research of this phenomenon in intubated and paralyzed subjects.

The ability of dynamic parameters to reflect volume status in mechanically ventilated patients and during NPPV has been demonstrated in previous studies [9, 16]. During spontaneous breathing the same parameters are less useful, dynamic flow parameters have however demonstrated an ability to reflect volume status in some studies [7, 28, 29], although only passive leg raise and end-expiratory occlusion test have shown convincing results [30, 31]. Our finding that Δ peak aortic flow was the best dynamic tracker of LBNP supports consideration of respiratory blood flow variation also in spontaneously breathing patients.

### Non-dynamic parameters

The observations regarding non-dynamic parameters are in agreement with our second hypothesis, that non-dynamic PWR parameters could track hypovolemia better than equivalent pressure and flow parameters. This is reasonable since the PWR-integral will incorporate both reductions in flow and pressure, but the pressure-reduction was small while the flow-reduction was substantial. One could imagine that oscillatory/pulsatile energy would change in hypovolemia [11], and since the PWR-integral incorporates both mean and oscillatory/pulsatile energy, it would not necessarily track hypovolemia better than SV and pulse pressure. One could also imagine the timing of the peak pressure and peak flow in each cardiac cycle shifting with hypovolemia. This would make the calculation of PWR_max_ unpredictable with increasing hypovolemia. These speculations are not supported by our findings.

### Clinical application

Evaluating hypovolemia and fluid responsiveness during spontaneous breathing with Δ peak aortic flow could be challenging in clinical practice. The cut-off value for hypovolemia seems to be around 12 % respiratory variation in Δ peak aortic flow, which may be difficult to distinguish from the baseline values of approximately 9 % respiratory variation using an ultrasound monitor. Of the non-dynamic parameters the PWR-integral tracks hypovolemia slightly better than SV, however considering the technical complexity in acquiring the PWR-integral today, SV seems adequate based on these results in healthy volunteers. If further research shows better results for power parameters in patient populations, a relatively small technological adaptation of ultrasound scanners would be necessary to make power parameter easily available. For now we would recommend paying attention to aortic flow and SV with ultrasound where hypovolemia is suspected, as the information provided may be valuable combined with other clinical signs of hypovolemia.

### Limitations of the study

The subjects in this study were healthy, young volunteers. The usefulness of power parameters could be better in older patients, and in patients with illnesses and conditions affecting the cardiovascular system, where blood pressure is likely to be more affected by hypovolemia. Further studies would be necessary to clarify this.

Blood pressure was measured using Finometer on the left third finger. A proper cardiac power calculation should be based on aortic pressure, which would demand methods too invasive to justify in healthy volunteers, and probably inapplicable in most clinical circumstances. Finometer has in many studies shown good agreement with radial pressure [25, 32]. Although radial pressure produces reliable measures of diastolic and mean aortic pressure, radial systolic pressure is often higher than aortic systolic pressure [33]. We only used time calibration as a transfer function from radial to aortic pressure, in the magnitude of 5–10 milliseconds, so that the systolic upstrokes in pressure and flow were synchronized before calculating the power curve. This probably introduced an error compared to cardiac power based on aortic pressure, as the systolic cardiac power value based on radial pressure often will result in an overestimation. As long as radial blood pressure is the standard for continuous blood pressure measurement in clinical use, it is however most clinically relevant to base cardiac power measurements on radial blood pressure.

The aortic diameter was assumed to be 20 mm in every individual as in previous studies involving this method of cardiac output determination [19]. When considering absolute values of all flow and cardiac power related parameters, this will introduce an error. However, when considering relative changes from baseline values due to increasing LBNP as in this study, an error in aortic diameter will not affect the result assuming a fairly constant diameter [20] in each individual. Similarly, errors introduced by assuming a 20 ^o^ angle would also be insignificant when considering relative changes due to LBNP, assuming the probe position is relatively constant in each individual.

This study used the suprasternal window to measure aortic flow. In clinical use an apical window may be more appropriate, but this window was obstructed by the LBNP-chamber in this study. Achieving aortic flow through the apical window is considered a relatively basic echocardiographic skill, achievable in most patients [34].

Using volume controlled ventilation in non-sedated subjects is unusual in clinical practice. All kinds of controlled ventilation in healthy, non-sedated subjects is challenging. One aim of the study was to compare dynamic variables during hypovolemia and positive pressure ventilation. Aortic flow variations and pulse pressure variations are induced by cyclic changes in intrathoracic pressure. These cyclic changes are induced by positive pressure ventilation and disturbed by any spontaneous breathing activity, including triggering. Pressure support would naturally lead to spontaneous breathing efforts. Pressure control mode (BiPAP) led to uneven tidal volumes and excessive spontaneous breathing efforts. Volume control (IPPV) yielded constant tidal volumes and minimized spontaneous breathing efforts. One possible reason could be that while the tidal volumes are kept constant during IPPV, the mode allows for variation in the inspiratory pressures required to obtain these volumes. Some subjects could not comply with controlled ventilation at all, and were not included in the study. After thorough testing before the experiments, we found volume control mode with relatively large tidal volumes (10 ml/kg) to minimize spontaneous breathing activity, which would have interrupted the cyclic intrathoracic pressure we aimed for. The physiological aspect of investigating respiratory variations in positive pressure ventilation with increasing hypovolemia simulated by LBNP was however achieved, allowing us to compare different dynamic parameters to each other.

The sample size is limited, but comparable to other LBNP-studies, and we were able to demonstrate several significant effects indicating sufficient statistical power.

## Conclusions

Dynamic power parameters were less able to track hypovolemia than dynamic flow- and pressure-parameters both during spontaneous ventilation and NPPV, seemingly due to a previously unknown phase difference between respiratory variations of pressure and flow. The PWR-integral was the best non-dynamic parameter in tracking hypovolemia during both spontaneous breathing and NPPV in healthy volunteers, however only slightly better than SV.

## Abbreviations

CO, cardiac output; CPO, cardiac power output; HR, heart rate; LBNP, lower body negative pressure; MAP, mean arterial pressure; NPPV, non-invasive positive pressure ventilation; Peak aortic flow, maximal aortic flow velocity in one cardiac cycle; PP, pulse pressure; PWR, cardiac power; PWR-integral, cardiac power integral; PWR_max_, maximal cardiac power in one cardiac cycle; SBP, systolic blood pressure; SV, stroke volume; Δ peak aortic flow, respiratory variation in maximal aortic flow velocity; Δ PP, respiratory variation in pulse pressure; Δ PWR-integral, respiratory variation in cardiac power integral; Δ PWR_max_, respiratory variation in maximal cardiac power; Δ SBP, respiratory variation in systolic blood pressure; Δ SV, respiratory variation in stroke volume
